# Metabolomic and Physiological Changes in *Fagus sylvatica* Seedlings Infected with *Phytophthora plurivora* and the A1 and A2 Mating Types of *P. ×cambivora*

**DOI:** 10.3390/jof8030298

**Published:** 2022-03-14

**Authors:** Tamara Corcobado, Ivan Milenković, Iñigo Saiz-Fernández, Tomáš Kudláček, Roman Plichta, Tomáš Májek, Aneta Bačová, Henrieta Ďatková, László Benedek Dálya, Miloš Trifković, Davide Mureddu, Vladimír Račko, Monika Kardošová, Jaroslav Ďurkovič, Roman Rattunde, Thomas Jung

**Affiliations:** 1 Phytophthora Research Centre, Faculty of Forestry and Wood Technology, Mendel University in Brno, 61300 Brno, Czech Republic; tamara.sanchez@mendelu.cz (T.C.); ivan.milenkovic@mendelu.cz (I.M.); tomas.kudlacek@mendelu.cz (T.K.); tomas.majek@mendelu.cz (T.M.); aneta.bacova@mendelu.cz (A.B.); xdatkova@mendelu.cz (H.Ď.); 2 Faculty of Forestry, University of Belgrade, Kneza Višeslava 1, 11030 Belgrade, Serbia; 3 Phytophthora Research Centre, Laboratory of Molecular Biology and Radiobiology, Faculty of AgriSciences, Mendel University in Brno, Zemědělská 1, 61300 Brno, Czech Republic; inigo.fernandez@mendelu.cz; 4 Department of Forest Botany, Dendrology and Geobiocoenology, Faculty of Forestry and Wood Technology, Mendel University in Brno, Zemědělská 3, 61300 Brno, Czech Republic; roman.plichta@mendelu.cz; 5 Department of Forest Protection and Wildlife Management, Faculty of Forestry and Wood Technology, Mendel University in Brno, Zemědělská 3, 61300 Brno, Czech Republic; laszlo.dalya@mendelu.cz (L.B.D.); milos.trifkovic@mendelu.cz (M.T.); 6 Department of Agricultural Sciences, University of Sassari, Viale Italia 39, 07100 Sassari, Italy; davidemureddu93@gmail.com; 7 Department of Wood Science, Technical University in Zvolen, 96001 Zvolen, Slovakia; racko@tuzvo.sk; 8 Department of Phytology, Technical University in Zvolen, 96001 Zvolen, Slovakia; kardosova@tuzvo.sk (M.K.); jaroslav.durkovic@tuzvo.sk (J.Ď.); 9 Institute of Botany, Technische Universität Dresden, 01062 Dresden, Germany; r-rattunde@online.de; 10 Phytophthora Research and Consultancy, Am Rain 9, 83131 Nußdorf, Germany

**Keywords:** root pathogens, mortality, gas exchange, spectral reflectance, chlorophyll fluorescence, photosynthesis, GABA, sugars, proline

## Abstract

*Phytophthora* infections are followed by histological alterations, physiological and metabolomic adjustments in the host but very few studies contemplate these changes simultaneously. *Fagus sylvatica* seedlings were inoculated with A1 and A2 mating types of the heterothallic *P. ×cambivora* and with the homothallic *P. plurivora* to identify plant physiological and metabolomic changes accompanying microscope observations of the colonization process one, two and three weeks after inoculation. *Phytophthora plurivora*-infected plants died at a faster pace than those inoculated with *P. ×cambivora* and showed higher mortality than *P. ×cambivora* A1-infected plants. *Phytophthora ×cambivora* A1 and A2 caused similar progression and total rate of mortality. Most differences in the physiological parameters between inoculated and non-inoculated plants were detected two weeks after inoculation. Alterations in primary and secondary metabolites in roots and leaves were demonstrated for all the inoculated plants two and three weeks after inoculation. The results indicate that *P. plurivora* is more aggressive to *Fagus sylvatica* seedlings than both mating types of *P. ×cambivora* while *P. ×cambivora* A1 showed a slower infection mode than *P. ×cambivora* A2 and led to minor plant metabolomic adjustments.

## 1. Introduction

*Phytophthora* species are serious pathogens of forest trees worldwide, causing a variety of disease symptoms and huge ecological and economical losses [1]. In Europe, typical examples of devastating *Phytophthora* epidemics of forest ecosystems are oak decline caused by *P. cinnamomi* Rands, *P. quercina* Jung and other species [1,2,3,4,5], beech decline caused by *P. plurivora* Jung and Burgess and *P. ×cambivora* Petri (Buisman) and other species [1,6,7,8,9], and alder dieback caused by species from the *P. ×alni* complex [1,10,11,12,13]. The biggest risk for the health and stability of European forests is the introduction of non-native pathogenic *Phytophthora* species from other continents into previously uninfected forest ecosystems via the planting of infested nursery stock [1,14].

One of the most widespread and economically and ecologically most important tree species in temperate regions of Europe is the European beech (*Fagus sylvatica* L.). However, this tree species is highly susceptible to *Phytophthora* infections and an extensive decline has been observed since the mid-1990s in natural forests, planted forests, parks and amenity trees [1,6,8,9]. *Phytophthora ×cambivora* and *P. plurivora* seem to be the most aggressive and most common *Phytophthora* spp. associated with declining beech, attacking fine and woody roots, the bark at the collar and along stems and branches, and shoots [1,6,9,15,16,17,18,19,20,21]. *Phytophthora ×cambivora*, previously known as *P. cambivora* and one of the causal agents of ink disease of chestnut [1,8,22], was recently redescribed as an allopolyploid hybrid species within *Phytophthora* Clade 7a which most likely originated from East Asia [17]. It has a heterothallic mating system (i.e. sexual reproduction only when the two compatibility (=mating) types A1 and A2 co-occur) and high intraspecific variability. It is commonly found as an invasive pathogen in the Americas and Europe. In the Americas the mating types of *P. ×cambivora* show regional differences in predominance. For instance, in Oregon, the A1 mating type is more frequently isolated from natural and semi-natural ecosystems than the A2 mating type [23,24], while in Argentina and Chile only the A2 type was isolated from soil samples collected under *Austrocedrus chilensis* and *F. sylvatica* trees, respectively [25,26]. In European natural ecosystems the A2 mating type of *P. ×cambivora* is apparently more widespread than the A1 mating type [17,19,21]. However, most studies did not specify the mating type. In pathogenicity trials with golden chinquapin (*Chrysolepis chrysophylla*) in both natural forests and in a greenhouse in Oregon, both mating types showed similar aggressiveness in terms of mortality and bark lesion area [23]. In other pathogenicity trials which tested either the A1 [24,27] or the A2 mating type of *P. ×cambivora* [17,21,28] bark lesions on stems and roots and root rot developed on different hosts. At a global scale there is a lack of pathogenicity tests comparing aggressiveness of both mating types of *P. ×cambivora*, or other heterothallic *Phytophthora* species in general, to native woody plants. This raises the question whether both mating types of *P. ×cambivora* are posing a similar threat to European tree species. The exotic invasive *P. plurivora* is also commonly associated with declining European beech forests [1,8,9,29] and demonstrated high aggressiveness to beech in pathogenicity tests [15,30,31]. It is a homothallic species within phylogenetic Clade 2c, also known as the ‘*P. citricola* species complex’, which most likely originated in southeast and east Asia [32,33]. 

On various host plant species the physiological effects of *Phytophthora* inoculations have been demonstrated including significant reductions in CO_2_ assimilation, stomatal conductance and chlorophyll fluorescence [34,35,36]. Only a few studies examined and showed changes in spectral reflectance at greenhouse [37] and field assays [38]. Physiological alterations in early infection stages include reduced photosynthetic rates which can be accompanied by simultaneous or subsequent hydraulic failures [39]. The study of metabolomics is also a powerful tool that provides deeper insights into the intra- and extracellular interactions of plant cells. Several studies have shown that numerous metabolites can discriminate *Phytophthora*-infected and non-infected plants [40,41]. However, there are no studies that combine both approaches, metabolomics and physiological assessments which could advance our understanding of pathogenesis. The objectives of this work were to (i) compare the aggressiveness of the A1 and A2 mating types of *P. ×cambivora* and *P. plurivora* on *F. sylvatica* seedlings, and (ii) assess the plant physiological and metabolomic alterations caused by the infections. 

## 2. Materials and Methods

### 2.1. Plant Material

Five months-old *F. sylvatica* seedlings grown from stratified seeds were used in the pathogenicity trial. Seeds were collected from a *F. sylvatica* seed plantation located in Jizerské Hory region in the north of Czech Republic. Seedlings were grown in 12 cell-seed trays containing perlite as substrate and placed in an airconditioned greenhouse at 20–22 °C with a light regime of 16 h light and 8 h darkness. The volume of each cell in the tray was approximately 220 cm^3^. Each tray was placed in a plastic box and randomly distributed in the greenhouse. Two months prior to the inoculation, plants were fertilized with granules of a NPK fertilizer (Osmocote, ICL, Ipswich, UK).

### 2.2. Inoculation

Four replicates of 12-cell trays per *P. ×cambivora* isolate with 10–11 *F. sylvatica* seedlings were used. Plants were inoculated in July 2019 with each two different isolates of the A1 and A2 mating types of *P. ×cambivora*, respectively. In addition, two replicates of 12-cell trays (in total 22 seedlings) were inoculated with an isolate of *P. plurivora* as a comparison. Five replicates of 12-cell trays with 10–11 *F. sylvatica* seedlings per tray were used as non-infested control. The isolates for this study were sourced from the Phytophthora Research Centre culture collection: TJ29 (*P. ×cambivora* A2 isolate from *F. sylvatica* in Slovakia), TJ197 (*P. ×cambivora* A2 neotype isolate CBS 141218 from *Quercus pubescens* in Italy), TJ30 and TJ543 (*P. ×cambivora* A1 isolates from *F. sylvatica* in Germany and Belgium, respectively) and TJ71 (*P. plurivora* extype isolate CBS 124093 from *F. sylvatica* in Germany). All isolates were collected by T. Jung except for the isolate TJ543 which was isolated (under the code Resi 75) by Anne Chandelier.

**Figure 1:**
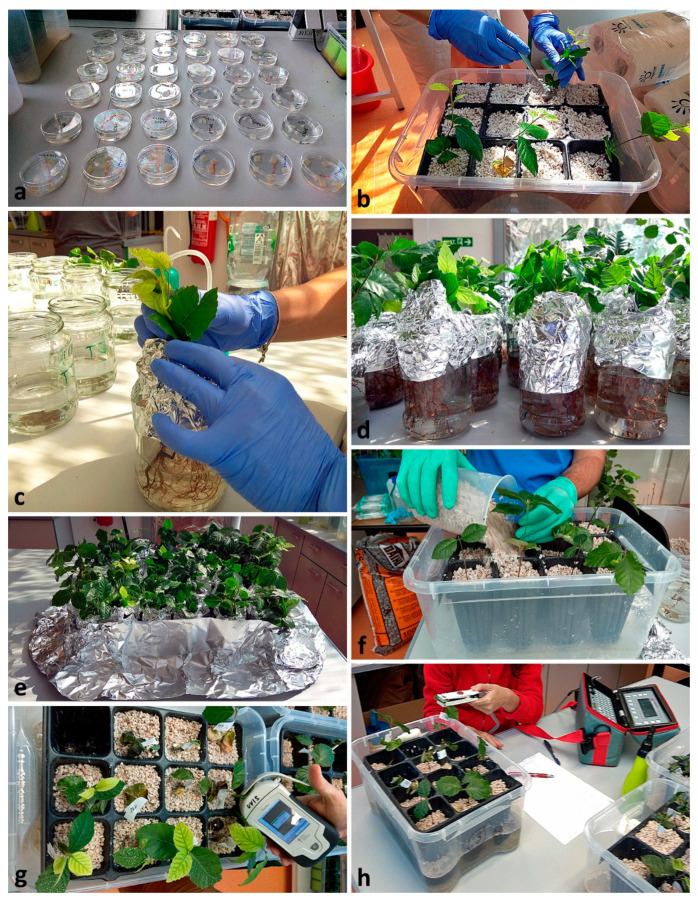
Methodology of the pathogenicity trial; (**a**) stimulation of *Phytophthora* sporangia production by flooding agar discs with active mycelium in distilled water; (**b**) extraction of *Fagus sylvatica* seedlings from the cell-trays; (**c**) immersion of the root tips into a jar containing distilled water and *Phytophthora* agar plugs with sporangia; (**d**,**e**) jars with the four *Fagus sylvatica* -*Phytophthora* isolate combinations plus the non-inoculated control subjected to 48 h of immersion; (**f**) *Fagus sylvatica* seedlings placed back to the cell trays; (**g**) weekly spectral reflectance assessments using a PolyPen; (**h**) weekly stomatal conductance measurements using a portable porometer.

The isolates were subcultured onto clarified V8-juice agar media (V8A), prepared with 100 mL/L of clarified vegetable juice (Pfanner Gmbh, Lauterach, Austria); 2 g/L of CaCO3; 18 g/L of agar (Sigma Group a.s., Lutín, Czech Republic); and 900 mL/L of distilled water. All isolates were incubated at 22–25 °C in the dark. Then mycelial agar squares of ca. 1.5 × 1.5 cm were cut with a sterilized scalpel from the margins of 6–8-day-old cultures of the respective *Phytophthora* isolates. The agar plugs were immersed in sterile 90 mm Petri dishes filled with distilled water (Figure 1a). Within the first six hours the water was changed twice and then the plugs were placed in sterilized 0.7 L glass jars. The beech seedlings were gently extracted from the cells (Figure 1b) and inoculated by immersion of their bare root systems in the jars containing 0.5 L of distilled water and five mycelial agar plugs of the respective *Phytophthora* isolates (Figure 1c,d). After 48 h of incubation (Figure 1e), the seedlings were potted again (Figure 1f) in the cell trays within the plastic boxes containing 2 L of distilled water and 15 agar plugs of the respective *Phytophthora* isolate. Before the inoculations, the sporangia production of all isolates on the plugs was confirmed under the light microscope at ×80 magnification. To check the ability of the plugs to sporulate, rebaitings were performed using young *Quercus suber* and *F. sylvatica* leaves as baits [6]. After 48 h the flooding water was removed, and normal drainage resumed. During the whole duration of the trial, the plants were incubated at 20 °C in an air-conditioned greenhouse.

### 2.3. Sampling for Microscopic Studies

**Figure 2:**
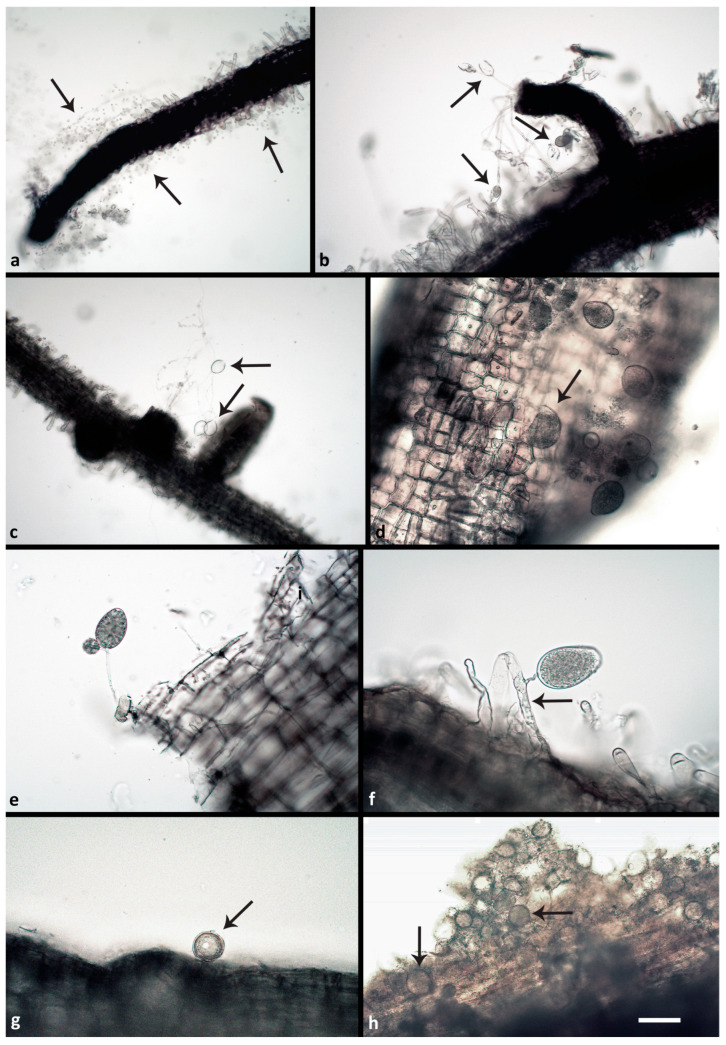
*Phytophthora* structures formed on the surface or inside of *Fagus sylvatica* fine roots two and three weeks post inoculation (p.i.) with *Phytophthora* zoospores: (**a**) zoospores and zoospore cysts of *P. ×cambivora* A2 (arrows) 2 weeks p.i.; (**b**) sporangia of *P. ×cambivora* A2 (arrows) emerging 2 weeks p.i. from an infected lateral root; one of them showing nested proliferation; (**c**) empty ovoid sporangia of *P. plurivora* (arrows) after zoospore release, emerging 2 weeks p.i. from an infected fine root; (**d**) numerous semipapillate and bipapillate (arrow) sporangia of *P. plurivora* formed 3 weeks p.i. on an infected fine root; (**e**) nonpapillate ovoid sporangium of *P. ×cambivora* A1 with external proliferation emerging 3 weeks p.i. from an infected fine root; (**f**): nonpapillate ovoid sporangium of *P. ×cambivora* A2 emerging 3 weeks p.i. from a root hair infected with a hypha (arrow); (**g**) globose oogonium, containing an oospore, of *P. plurivora* (arrow) formed on the surface of an infected fine root 2 weeks p.i.; (**h**) numerous immature oogonia of *P. plurivora* (arrows) produced inside an infected fine root 3 weeks p.i.

One, two and three weeks after the inoculation, each 2–3 plants per *F. sylvatica*-*Phytophthora* isolate combination and control were removed from the pots and the perlite gently washed out from the roots. Roots were surface-dried on sterile filter paper and kitchen towels, and a photo documentation was created (Figure 2a–h). Five to eight lateral root and taproot segments per plant were harvested and placed on a glass slide. Each root segment was then examined under the light microscope (Motic^®®^, Wetzlar, Germany) at ×40, ×100, and ×400 magnifications to determine the presence of hyphae and spores of *P. ×cambivora* and *P. plurivora* on the root surface and inside the root tissue. Roots of control plants were also examined. 

At the end of the experiment after 3 weeks, additional root fragments were sampled from surviving plants and subjected to scanning electron microscopy. Root segments sampled from both tap roots and root collar were fixed overnight in FAA solution (formaldehyde: glacial acetic acid: ethanol, 1: 1: 18 *v*/*v*/*v*) at 4 °C. Free-hand transverse sections were dehydrated in ethanol and acetone, and dried in liquid CO2 using a Leica EM CPD030 critical point drier (Leica Microsystems, Wetzlar, Germany). Root sections were mounted on specimen stubs, sputter-coated with gold in the Sputter Coater K650X (Quorum Technologies, Ashford, UK) under argon atmosphere, and examined by high-vacuum scanning electron microscopy using a JEOL JSM-6390 instrument (JEOL, Tokyo, Japan) operating at the range of 15 to 20 kV.

### 2.4. Re-Isolation of Phytophthora spp.

Simultaneously with the sampling for microscopic assessments one, two and three weeks after the inoculation, 10–15 necrotic root samples were taken from each 2–3 plants per *F. sylvatica*-*Phytophthora* isolate combination and the control and plated onto selective PARPNH agar [2] to confirm the presence of the inoculated *Phytophthora* species and, hence, fulfilling Koch’s postulates. Hyphae growing from plated root fragments were subcultured onto V8A and incubated at 20 °C in the dark. Classical morphological species identification at ×400 magnification under a light microscope was performed according to [29,32].

### 2.5. Physiological Measurements

A minimum subset of six plants per *F. sylvatica*-*Phytophthora* isolate combination was randomly selected for the physiological measurements (Figure 1g,h). Starting from the plant apex two to six fully expanded leaves were used for the physiological measurements, with readings recorded from the adaxial leaf surface. Measurements were taken one, two and three weeks after inoculation. Leaf stomatal conductance (gs) measurements were taken with a portable porometer (AT porometer AP4, Delta-t devices, Burwell, Cambridge, UK) from 12:00 to 15:30. Steady-state maximum quantum yield of photosystem II (Fv/Fm) and leaf reflectance were measured using FluorPen FP100 and PolyPen RP 400 UVIS instruments, respectively (Photon Systems Instruments, Brno, Czech Republic) from 9:00 to 11:30. Four reflectance-based physiological indices that are based on chlorophyll and carotenoids absorption features were determined from the PolyPen dataset: the Normalized Difference Vegetation Index (NDVI) = (R780-R630)/(R780 + R630), [42], the Photochemical Reflectance Index (PRI) = (R531 − R570)/(R531 + R570) [43], the Structure Intensive Pigment Index (SIPI) = (R790 − R450)/(R790 + R650) [43] and Carotenoid Reflectance Index (CRI1) = 1/R510 − 1/R550 [44], where R refers to reflectance and subscript indicates the wavelength in nanometers.

### 2.6. Metabolomic Analyses 

A total of six plants from the *P. plurivora* inoculation treatment and 12 plants from the *P. ×cambivora* inoculation and control treatments were collected for the metabolomics analysis 2 and 3 weeks after inoculation. Plants were gently removed from their cells and leaf and fine root samples were collected, immediately frozen, and kept at −80 °C until further processing. After homogenization, approximately 0.1 g of fresh weight were extracted using the methanol/ether/water method, as described previously [45]. Briefly, 1 mL of methanol/methyl tert-butyl ether/water (1:3:1) was added to each sample, which were then kept at 4 °C for 30 min under gentle shaking. Afterwards, 0.5 mL of methanol/water (1:3) were added and the samples were centrifuged to allow phase-separation. The apolar phase was collected and 30 µL were aliquoted for the metabolomic analysis.

For sample derivatization, aliquots of the apolar phase were vacuum-dried, 20 µL of 40 mg/mL methoxyaminhydrochloride (dissolved in pyridine) were added, and samples were incubated for 90 min at 30 °C. Then, 80 µL of MSTFA were added and samples were incubated for further 30 min at 37 °C. Lastly, samples were centrifuged and around 50 µL were transferred into glass inserts prior to injection in the GC-MS. Derivatized samples were injected in a Thermo-Fisher Q Exactive GC Orbitrap GC-MS for untargeted full scan profiling. The resulting chromatograms were processed using Thermo TraceFinder software, with which peak retention times and ion masses were compared to those covered in Mainlib and GC-Orbotrap Metabolomics libraries. The areas of those peaks with positive identification (score ≥75 and ΔRI <5%) were compared to the equivalent peaks in control samples in order to visualize the changes in plant metabolome following *Phytophthora* infection. A minimum of six independent biological replicates were performed per time and isolate/mating type.

### 2.7. Data Analysis

#### 2.7.1. Mortality

Survival curves describing the relationship between the mortality rate and time after the infection were computed using the Kaplan–Meier method implemented in the R package survival [46]. Differences in the survival curves between the plants infested with the corresponding mating types/species and between the plants infested with the corresponding mating types/isolates and the non-infested plants were analyzed by means of log-rank test with a correction for multiple testing using the R package survminer [47]. The differences in mortality rates at the end of the experiment (3 weeks after the inoculation) between the plants inoculated with the different mating types, species and isolates of *Phytophthora* and between the plants inoculated with the different mating types, species and isolates of *Phytophthora* and the non-infected plants were tested using the generalized linear model with the binomial family and subsequent likelihood ratio test using standard R functions [48] from the package stats.

#### 2.7.2. Physiology

All statistical analyses related to physiological parameters and mortality were performed in R 3.6.3 [48]. All tests were carried out at the significance level α = 0.05. Due to the lack of significant differences in mortality and in most physiological parameters between isolates within the same species and within the same mating type (Figure S1), all plants inoculated with the same mating type of *P. ×cambivora* were considered as one treatment. Plants that were found dead and could therefore not be measured 3 weeks after the inoculation were excluded from the dataset. 

Differences in the leaf conductance, maximum quantum yield of photosystem II and spectral reflectance indices between the mating types/individual isolates and non-infected plants were analyzed by means of generalized linear models (GLM) and follow-up likelihood ratio tests (LRT) using standard R functions from the package stats. Multiple comparisons were conducted via the R package multcomp [49] using the HC3 estimator accounting for heterogeneous variances implemented in the R package sandwich [50,51,52].

#### 2.7.3. Metabolomic Profiling

The metabolomic differences between treatments were analyzed by means of log-normal generalized linear models followed by the likelihood ratio test and multiple comparisons as described above. Heatmaps were constructed using the R package ComplexHeatmap [53]. Values displayed in the heatmaps are log base 2 of the ratio between the peak metabolite areas of the tested samples and the respective reference groups.

#### 2.7.4. Visualization

The figures accompanying all the above-mentioned analyses were created with R packages ggplot [54] and survminer [47] and Inkscape 0.92 [55].

## 3. Results

### 3.1. Viability of Isolates and Light and Scanning Electron Microscopy Observations

**Figure 3:**
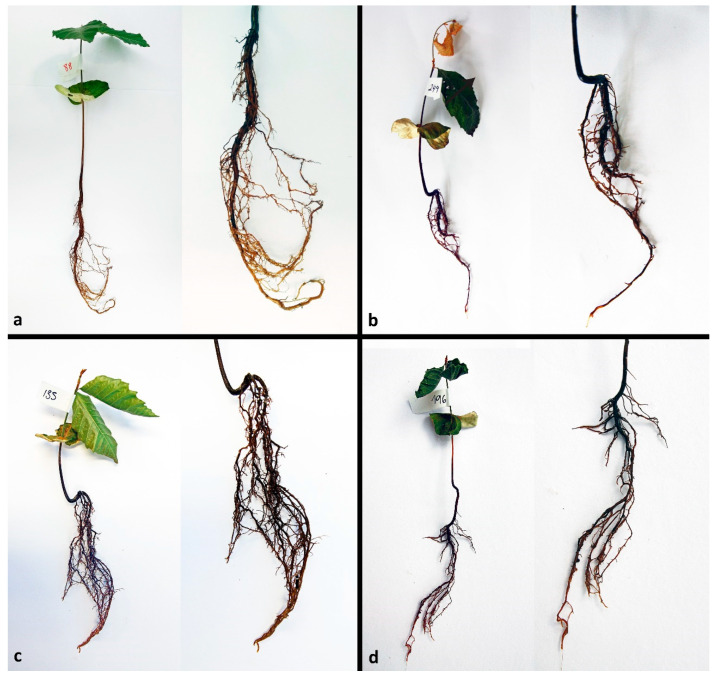
Representative *Fagus sylvatica* seedlings and their root systems three weeks after inoculation with *Phytophthora* zoospores; (**a**) non-inoculated control plant with healthy leaves and healthy root system with bright yellowish lateral roots and taproot; (**b**) wilting dying plant inoculated with *P. plurivora*; the taproot and most lateral roots are dark-brown and necrotic; (**c**) wilting plant inoculated with *P. ×cambivora* A1; the taproot and most lateral roots are dark-brown and necrotic; (**d**) wilting plant inoculated with *P. ×cambivora* A2; the taproot and most lateral roots are dark-brown and necrotic.

**Figure 4:**
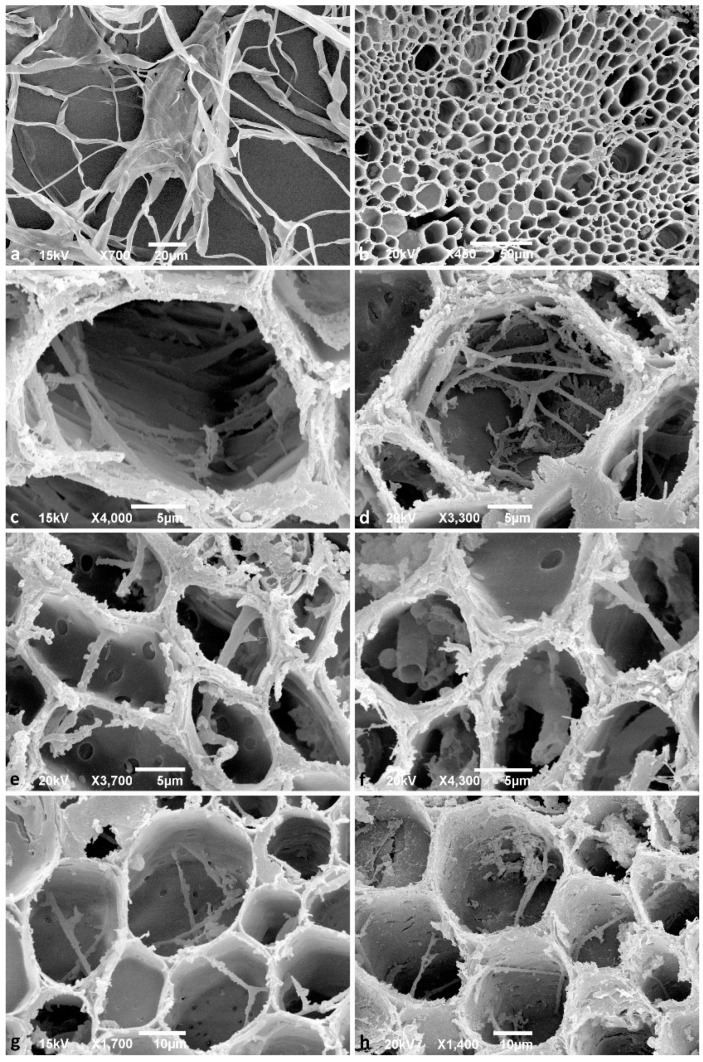
Scanning electron microscopy images. (**a**) Hyphae of *Phytophthora ×cambivora* A2 isolate TJ197 growing on V8-agar medium; scale bar = 20 μm; (**b**–**h**) cross-sections of *Fagus sylvatica* fine roots three weeks after inoculation with *Phytophthora* zoospores; (**b**) taproot of non-inoculated healthy control plant without any hyphae; scale bar = 50 μm; (**c**) hyphae of *P. ×cambivora* A1 isolate TJ30, spreading through a secondary xylem vessel of a taproot; scale bar = 5 μm; (**d**) hyphae of *P. ×cambivora* A1 isolate TJ543 in a parenchyma cell of the secondary xylem in a tap root; scale bar = 5 μm; (**e**) hyphae of *P. ×cambivora* A2 isolate TJ29 spreading via pits through parenchyma cells of the secondary xylem in a tap root; scale bar = 5 μm; (**f**) tubular thick hyphae of *P. ×cambivora* A2 isolate TJ197 inside parenchyma cells of the secondary xylem in a tap root; scale bar = 5 μm; (**g**) thinner hyphae of *P. plurivora*, isolate TJ71 spreading via pits through parenchyma cells of the secondary xylem in a tap root; scale bar = 10 μm; (**h**) hyphae of *P. plurivora* isolate TJ71 spreading via pits through parenchyma cells at the root collar; scale bar = 10 μm.

All respective *Phytophthora* isolates could be successfully reisolated from all plated root pieces. Furthermore, all isolates were successfully re-isolated from leaf baits floating in the water during the flooding period. One week after the inoculation (post inoculation = p.i.), zoospore cysts were abundantly observed on the root surfaces (Figure 2a), while sporangia were rare and scattered. Numerous cysts were germinating with germ tubes that penetrated the fine root epidermis. Two weeks p.i., microscopic observations of the roots revealed abundant production of sporangia and zoospores by the A2 mating type of *P. ×cambivora* (Figure 2b), and by *P. plurivora* (Figure 2c). Sporangia were produced on sporangiophores emerging from the surface of roots and fine root tips (Figure 2b,c), and even on the surface of fine root hairs, after the previous formation of appressoria-like structures (Figure 2a). *Phytophthora plurivora* had formed immature oogonia on the root surface (Figure 2g) and inside the tissue of lateral roots. However, on the surface of roots inoculated with the A1 mating type of *P. ×cambivora,* only cysts and hyphae could be observed. After 3 weeks the number of sporangia and germinating cysts on the roots of *P. plurivora*-infected plants had increased (Figure 2d). Moreover, the A1 mating type isolates of *P. ×cambivora* produced sporangia on the surface of taproots (Figure 2e) and lateral roots. The A2 mating type of *P. ×cambivora* also formed sporangia on the surface of fine root hairs (Figure 2f). Numerous oogonia of *P. plurivora* were observed inside necrotic root tissue (Figure 2h). After 3 weeks control plants had only healthy roots (Figure 3a) without the presence of any *Phytophthora* structures on the surface. In contrast, most taproots and lateral fine roots inoculated three weeks before with zoospores of the three *Phytophthora* species and mating types, respectively, were necrotic (Figure 3b–d). Examination of root fragments under the scanning electron microscope at the end of the experiment also showed the colonization of root tissues by all *Phytophthora* isolates. *Phytophthora* hyphae were found predominantly in taproots and at a lower density also in the root collar. In both root regions, hyphae had penetrated through the root cortex and colonized the internal root tissues by intracellular growth in the secondary xylem vessels and in parenchyma cells using the pits to spread between cells (Figure 4a,c–h). Control roots contained no hyphae (Figure 4b).

### 3.2. Survival Analysis 

**Figure 5:**
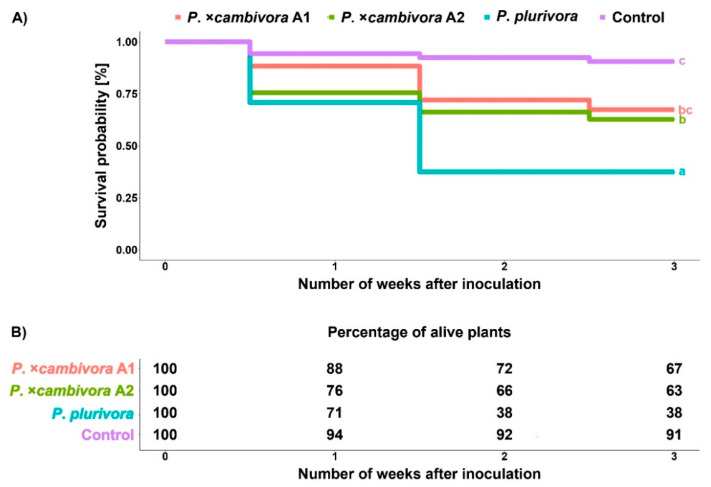
Mortality: (**A**) Survival probabilities of *Fagus sylvatica* seedlings three weeks after inoculation with *Phytophthora plurivora* and the A1 and A2 mating types of *Phytophthora* × *cambivora* and non-infected control plants using the Kaplan–Meier test; (**B**) percentage of living plants one, two and three weeks after inoculation.

*Fagus sylvatica* seedlings inoculated with *P. plurivora* were dying significantly faster than those inoculated with both mating types of *P. ×cambivora* (log-rank test, A1: *p* ≤ 0.01, A2: *p* ≤ 0.05) and control plants (log-rank test, *p* ≤ 0.001) (Figure 5A). The mortality was significantly higher and progressed faster for plants inoculated with either mating type of *P. ×cambivora* compared to control plants (A1: *p* ≤ 0.01, A2: *p* ≤ 0.001). The relationship between mortality rate and time after the inoculation did not differ significantly between the two mating types of *P. ×cambivora*, i.e., the mortality progressed at a similar pace. At the end of the experiment, the mortality rate of all inoculated plants was significantly higher compared to the control (Figure 5B; GLM + multiple comparison test, *P. ×cambivora* A1: *p* ≤ 0.05, *P. ×cambivora* A2: *p* ≤ 0.01, *P. plurivora*: *p* ≤ 0.001). Plants infected with *P. ×cambivora* A1 showed similar mortality rate (33%) as the plants infected with *P. ×cambivora* A2 (37%). There was a significant difference in mortality rate between *P*. ×cambivora A1 (33%) and *P. plurivora*-infected plants (62%) (GLM + multiple comparison test, *p* ≤ 0.05) and there was not a significant difference between *P. ×cambivora* A2 (37%) and *P. plurivora*-infected plants (62%) (GLM + multiple comparison test, *p* > 0.05).

### 3.3. Physiological Measurements

**Figure 6:**
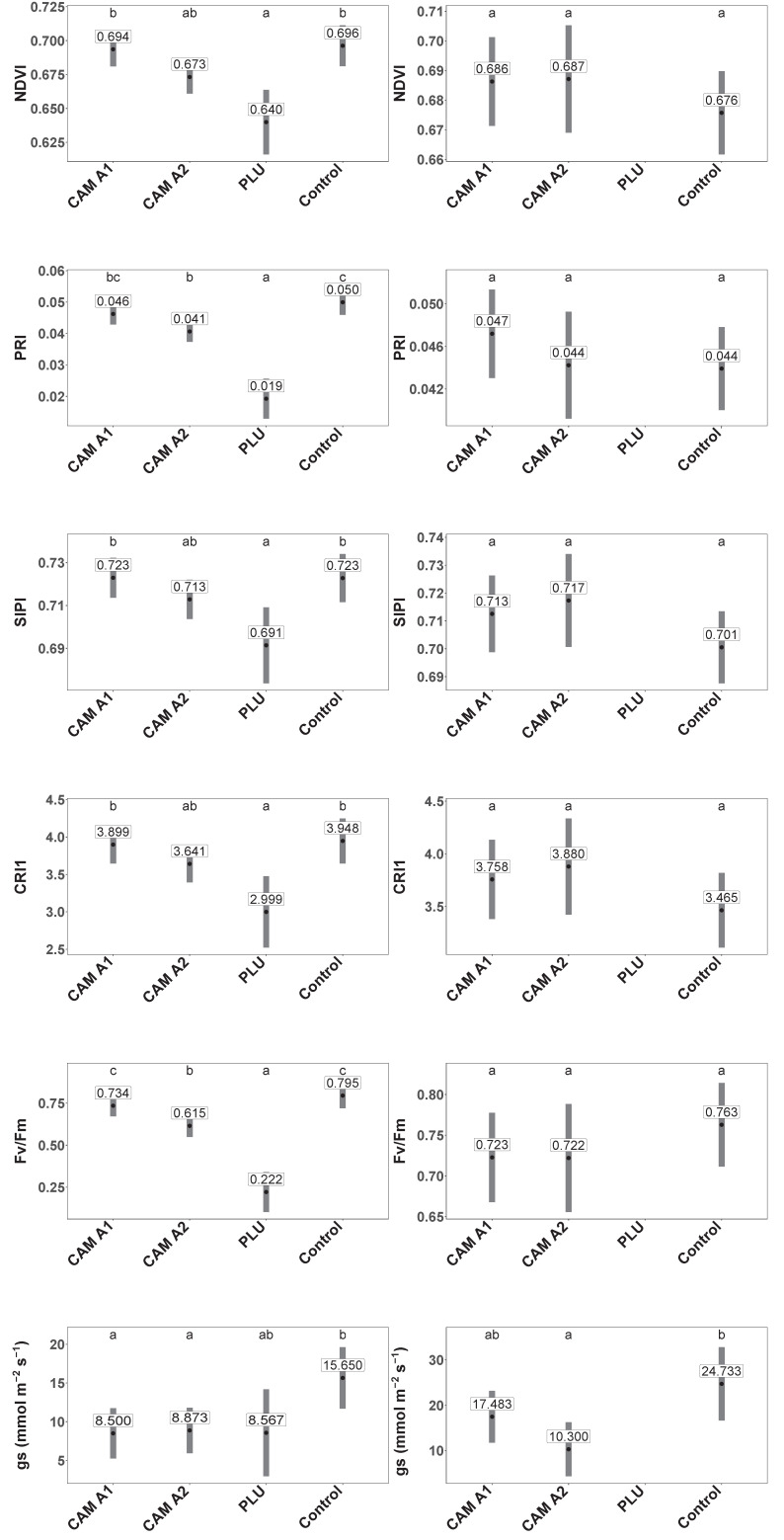
Mean physiological values of *Fagus sylvatica* seedlings infected with *Phytophthora plurivora* and the A1 and A2 mating types of *Phytophthora ×cambivora* and of non-infected control seedlings: Mean values of spectral reflectance calculated with the Normalized Difference Vegetation Index (NDVI), the Photochemical Reflectance Index (PRI), Structure Intensive Pigment Index (SIPI) and the Carotenoid Reflectance Index (CRI1), mean values of chlorophyll fluorescence expressed as maximum quantum yield of photosystem II and stomatal conductance values (gs) of the seedlings two and three weeks after inoculation. Different letters indicate statistical differences at significance level α = 0.05.

One-week p.i. *Phytophthora*-infected plants did not differ in any physiological parameter from control plants (GLM + multiple comparison test, *p* ≥ 0.05). However, two weeks after inoculation the *P. plurivora*-infected plants showed significantly lower values of all tested spectral reflectance indices and significantly lower value of F_v_/F_m_ compared to control plants (Figure 6; GLM + multiple comparison test, NDVI: *p* ≤ 0.001, PRI: *p* ≤ 0.001, SIPI: *p* ≤ 0.01, CRI1: *p* ≤ 0.01, F_v_/F_m_: *p* ≤ 0.001). The plants inoculated with *P. ×cambivora* A1 showed significantly lower g_s_ compared to control plants (Figure 6; GLM + multiple comparison test, *p* ≤ 0.05). Compared with the control, the plants inoculated with *P. ×cambivora* A2 showed a significantly lower value of the spectral reflectance index PRI (GLM + multiple comparison test, *p* ≤ 0.01), Fv/Fm (GLM + multiple comparison test, *p* ≤ 0.01) and g_s_ (GLM + multiple comparison test, *p* ≤ 0.01) (Figure 6). *Phytophthora plurivora*-infected plants showed significantly lower values of NDVI, PRI, SIPI, CRI1 and F_v_/F_m_ compared to *P. ×cambivora* A1-infected plants (GLM + multiple comparison test, NDVI: *p* ≤ 0.001, PRI: *p* ≤ 0.001, SIPI: *p* ≤ 0.001, CRI1: *p* ≤ 0.001, F_v_/F_m_: *p* ≤ 0.001) and significantly lower values of PRI and F_v_/F_m_ compared to *P. ×cambivora* A2-infected plants (GLM + multiple comparison test, PRI: *p* ≤ 0.001, F_v_/F_m_: *p* ≤ 0.001). The two mating types of *P. ×cambivora* did not differ from each other in any of the analyzed physiological parameters. Within *P. ×cambivora* A1, the isolates TJ30 and TJ543 showed similar effects on plants compared to control plants, except that TJ30 triggered a lower g_s_ after two weeks. Likewise, within *P. ×cambivora* A2 isolates TJ29 and TJ197 produced similar effects except for F_v_/F_m_, being lower in plants inoculated with TJ197 than in control plants after two weeks. 

Three weeks p.i., the mortality of *P. plurivora*-infected plants was already so high that there were not enough plants to carry out physiological measurements. The other isolates did not differ significantly between each other in any of the tested parameters with the only exception of the significantly lower value of g_s_ in *P. ×cambivora* A2-infected plants compared to the control (GLM + multiple comparison test, *p* ≤ 0.01). Within *P. ×cambivora* A1, both isolates resulted in a similar physiological status of plants. The same was observed in the plants inoculated with either of the isolates of *P. ×cambivora* A2.

### 3.4. Root and Leaf Metabolomic Profiling

**Figure 7:**
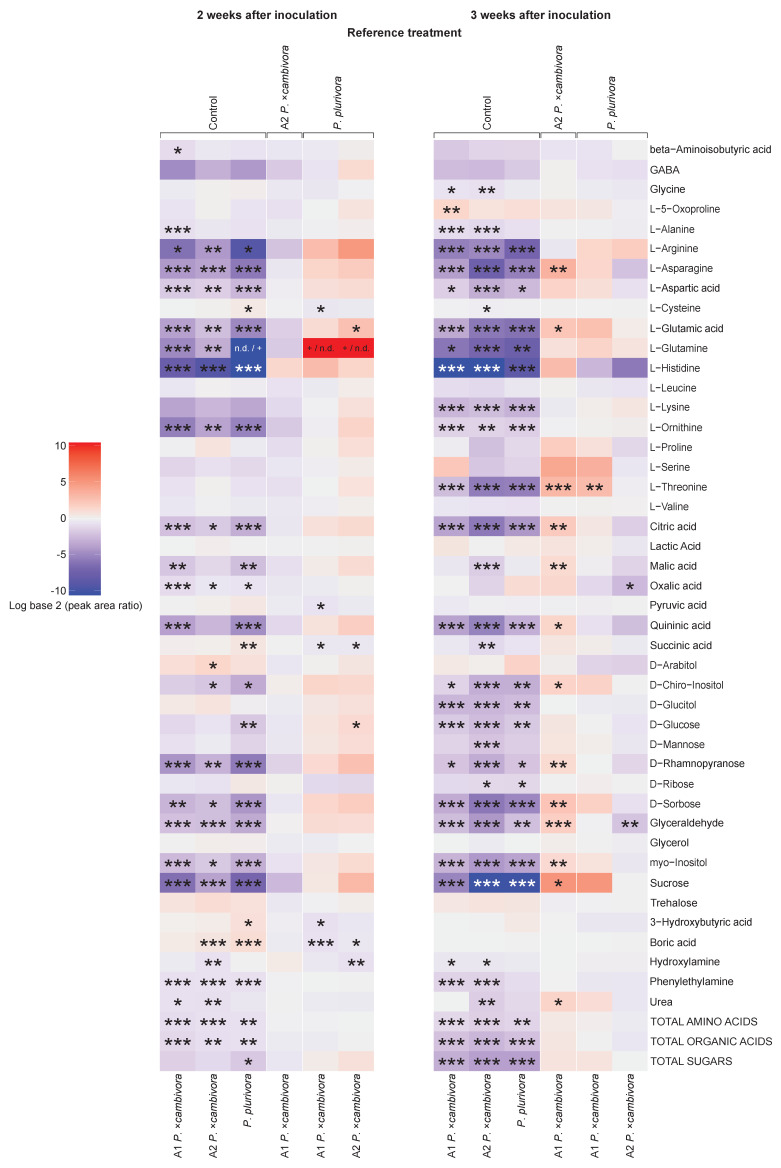
Heat map of primary and secondary metabolites identified in the roots of *Fagus sylvatica* seedlings inoculated with *Phytophthora plurivora* and the A1 and A2 mating types of *Phytophthora ×cambivora*. Different levels of concentration to control plants are depicted by different colors and their intensities (higher—red, lower—blue). Summaries of general metabolite groups (amino acids, organic acids, sugars) are shown next to each heatmap column. Statistical levels of significance: * = *p* ≤ 0.05, ** = *p* ≤ 0.01, *** = *p* ≤ 0.001.

In general, the metabolomic profiling revealed that the concentration of most identified metabolites in the roots of inoculated plants drastically decreased already two weeks after inoculation compared to control plants, regardless of the *Phytophthora* species and mating type (Figure 7). There was a significant decrease in the total amino acid concentration and total organic acid concentration in the roots of all inoculated plants 2 weeks p.i., which became even more pronounced after 3 weeks. In addition, the total sugar content was significantly lower in the roots of *P. plurivora*-infected plants compared to the control 2 weeks p.i., and in all inoculation treatments 3 weeks p.i. In particular, both the concentration and the relative abundance of sucrose were significantly decreased in all infected plants (Table S1). Interestingly, the concentration and relative abundance of sugar alcohols, i.e. D-glucitol, glycerol and L-arabitol, was not decreased in infected plants, and in some cases, it was even significantly higher than in roots of control plants. However, 3 weeks p.i. the differences to the controls were less pronounced and, in the case of D-glucitol, the concentration was significantly lower in infected plants. As an exception to the general trend observed in roots, L-5-Oxoproline increased significantly in plants inoculated with the A1 mating type of *P. ×cambivora* compared to control plants 3 weeks p.i. (GLM + multiple comparison test, *p* ≤ 0.01). 

**Figure 8:**
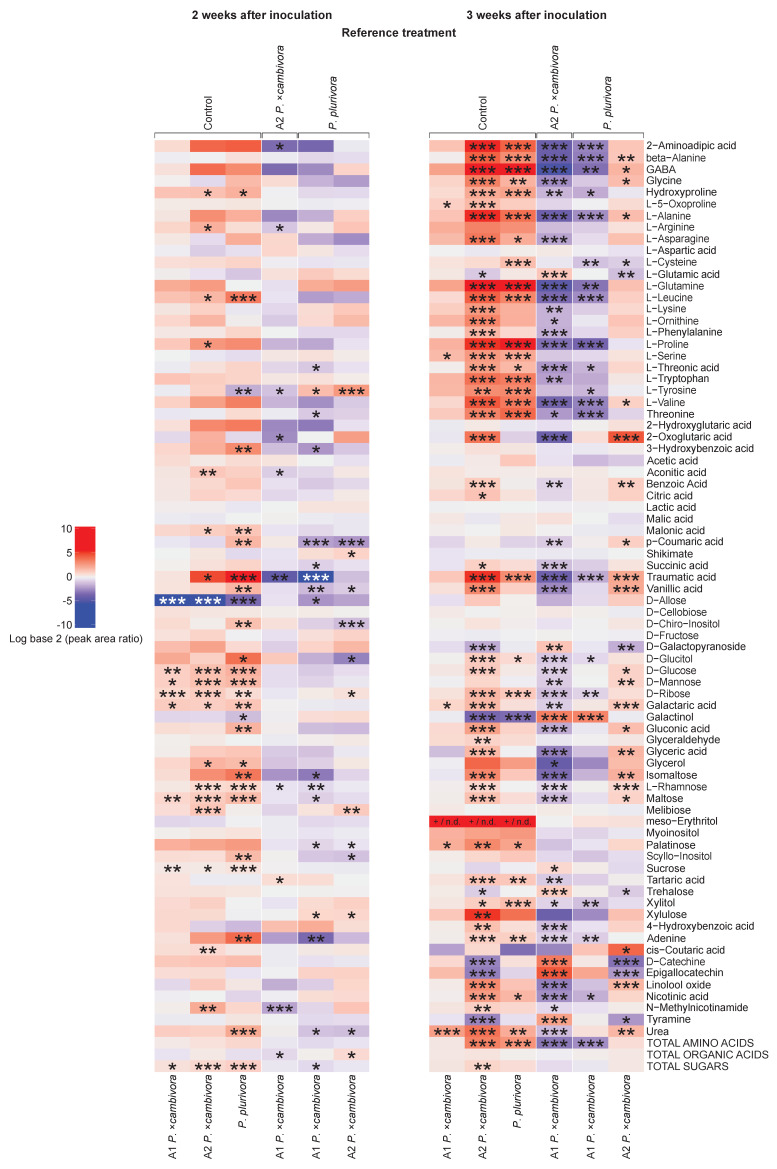
Heat map of primary and secondary metabolites identified in the leaves of *Fagus sylvatica* seedlings inoculated with *Phytophthora plurivora* and the A1 and A2 mating types of *Phytophthora ×cambivora*. Different levels of concentration to control plants are depicted by different colors and their intensities (higher—red, lower—blue). Summary of general metabolite groups (amino acids, organic acids, sugars) are shown next to each heatmap columns. Statistical levels of significance: * = *p* ≤ 0.05, ** = *p* ≤ 0.01, *** = *p* ≤ 0.001.

In the leaves, less pronounced differences were observed between infected and control plants, with an overall increase in metabolite concentrations in *Phytophthora*-infected plants (Figure 8). In particular, a significantly higher total sugar content was found in the leaves of all infected plants compared to control plants 2 weeks p.i. and in *P. ×cambivora* A2-infected plants also 3 weeks p.i. (Table S1). Differences in the metabolomic profiles of the infected plants between both mating types of *P. ×cambivora* and between both *Phytophthora* species were more obvious 3 weeks p.i. than 2 weeks p.i. and more pronounced in the leaves than in the roots. Two weeks p.i. the leaves of *P. ×cambivora* A1-infected plants displayed a significantly lower concentration of total organic acids compared to plants infected with *P. ×cambivora* A2 (GLM + multiple comparison test, *p* ≤ 0.05), and a significantly lower total sugar content compared to *P. plurivora*-infected plants (GLM + multiple comparison test, *p* ≤ 0.05). Leaves from plants infected with *P. ×cambivora* A2 showed a significantly higher total organic acid content than leaves from *P. plurivora*-infected plants (GLM + multiple comparison test, *p* ≤ 0.05) 2 weeks after the inoculation. Three weeks p.i. the leaves of *P. ×cambivora* A1-infected plants had a significantly lower concentration of total amino acids compared to plants infected with *P. ×cambivora* A2 (GLM + multiple comparison test, *p* ≤ 0.001) or *P. plurivora* (GLM + multiple comparison test, *p* ≤ 0.001). 

The level of L-proline, a well-known stress marker, was significantly increased in leaves of *P. ×cambivora* A2-infected plants compared to control plants 2 and 3 weeks p.i. (GLM + multiple comparison test, *p* ≤ 0.05 and *p* ≤ 0.001, respectively). Furthermore, *P. plurivora*-infected plants showed significantly higher leaf levels of this compound compared to control plants three weeks p.i. (GLM + multiple comparison test; *p* ≤ 0.001). In contrast, in leaves of plants infected with *P. ×cambivora* A1, L-proline levels did not differ significantly from the control and were significantly lower compared to plants infected with *P. ×cambivora* A2 (GLM + multiple comparison test; *p* ≤ 0.001) and *P. plurivora* (GLM + multiple comparison test; *p* ≤ 0.001) three weeks after inoculation.

Traumatic acid, a secondary metabolite related to plant physical damage, was significantly increased in leaves of plants infected with *P. ×cambivora* A2 and *P. plurivora* compared to control leaves both two (GLM + multiple comparison test: *P. ×cambivora* A2, *p* ≤ 0.05; *P. plurivora*, *p* ≤ 0.001) and three weeks p.i. (GLM + multiple comparison test: *P. ×cambivora* A2, *p* ≤ 0.001; *P. plurivora*, *p* ≤ 0.001). At both time points the concentration of traumatic acid was significantly lower in the leaves of plants infected with *P. ×cambivora* A1 compared to *P. ×cambivora* A2 (GLM + multiple comparison test: 2 weeks p.i., *p* ≤ 0.01; 3 weeks p.i., *p* ≤ 0.001) and *P. plurivora* (GLM + multiple comparison test: 2 weeks p.i., *p* ≤ 0.001; 3 weeks p.i., *p* ≤ 0.001). Three weeks after inoculation, traumatic acid was significantly higher in leaves of plants infected with *P. ×cambivora* A2 than in leaves of plants infected with *P. plurivora* (GLM + multiple comparison test; *p* ≤ 0.001). Overall, the biggest metabolic changes observed in leaves two and three weeks p.i. corresponded to *P. plurivora* and *P.* *×cambivora* A2, respectively, while *P.* *×cambivora* A1 showed the least differences compared to the controls at both time points. 

## 4. Discussion

The present study compared the effects of infections by *P. plurivora* and the A1 and A2 mating types of *P. ×cambivora* on *F. sylvatica* seedlings. To our knowledge, this is the first time that plants inoculated with *Phytophthora* pathogens were assessed simultaneously at physiological and metabolomic levels at different time points. Previous pathogenicity tests on one-year-old seedlings of *F. sylvatica* have shown within 3–5 months high aggressiveness of both pathogens, with *P. ×cambivora* causing on average 84–100% root rot and 20–90% mortality [15,17] and with *P. plurivora* (referred to as *P. citricola*) causing 88–90% root rot and 80–90% mortality [15,30,31]. Interestingly, in this pathogenicity trial despite causing similar mortality rates after 3 weeks, the two mating types of *P. ×cambivora* showed behavioral differences. Compared to the A2 type, the A1 type had a delayed sporangia production at the surfaces of the infected roots, which resulted in a later inoculum build-up, and was most likely responsible for the lower mortality rate one week after inoculation compared to the A2 mating type. The A1 mating type of *P. ×cambivora* also had weaker effects on the maximum efficiency of PSII after two weeks and on the metabolomic profile of leaves and to a lesser extent of roots three weeks after inoculation compared to the A2 mating type. In addition, the physiological responses (except stomatal conductance) and the metabolite concentration in roots of plants infected with *P. ×cambivora* A1 did not differ from control plants. In contrast, seedlings infected with *P. ×cambivora* A2 showed significant differences compared to the control in terms of PRI, maximum efficiency of PSII, stomatal conductance and metabolomics of roots, especially three weeks after inoculation, and metabolomics of leaves demonstrating subtle differences between the A1 and A2 mating types. *Phytophthora plurivora* was causing the fastest and highest mortality rates followed by *P. ×cambivora* A2 and *P. ×cambivora* A1. However, physiological reactions of *P. plurivora*-infected plants were not more pronounced than for *P. ×cambivora* A2. Similarly, differences in the metabolomic profiles of roots between plants infected with *P. plurivora* and plants infected with both mating types of *P. ×cambivora* were not noticeable. Only in leaves, the concentration of several metabolites in *P. plurivora*-infected plants differed statistically from *P. ×cambivora* A1 and A2. It is expected that if sampling for metabolomics would have been performed one week after the inoculation, the metabolomic profiles of *P. plurivora*-infected plants would have shown higher differences compared to plants infected by *P. ×cambivora* A1 and A2, especially in roots. However, two weeks after inoculation all infected plants had already developed the characteristic metabolomic adjustment mechanisms as a consequence of the *Phytophthora* attacks and no significant differences were observed between the more aggressive beech pathogen *P. plurivora* and *P. ×cambivora*.

There are different approaches to assess the physiological status of a plant, one of them being the calculation of spectral reflectance indices, which are complementary indicators of the status of the photosynthetic pigments (chlorophyll, xanthophyll and carotenoids) [56]. Lower values of the spectral reflectance indices of inoculated plants observed in our trial after two weeks are in agreement with other studies on different diseases such as Verticillium wilt on *Capsicum annuum*, Laurel wilt caused by *Raffaelea lauricola* on *Persea americana* or Cercospora disease on *Beta vulgaris* ssp. *vulgaris*, all showing a relationship between pathogen infections and a decrease of spectral indices [57,58,59]. In addition, [38] observed reduced reflectance indices of native Australian plants (i.e. *Banksia serrata*, *Dianella revoluta*, *Eucalyptus piperita* and *Lomandra longifolia*) infected with *P. cinnamomi*, but these differences only became significant 64 days after inoculation. In our study, the PRI index of *P. ×cambivora* A2-infected *F. sylvatica* seedlings and the NDVI, PRI, SIPI and CRI1 of *P. plurivora*-infected seedlings were already reduced two weeks after inoculation demonstrating that several spectral reflectance indices can be used for the early detection of root infections in certain pathosystems. However, infections by the A1 mating type of *P. ×cambivora* did not result in the decrease of any spectral reflectance indices. Whether other spectral indices, which consider different wavelength ranges, would show alterations in plants affected by *P. ×cambivora* A1, hence, enabling the detection of this pathogen, remains unknown and would require further research. 

Together with changes in spectral indices, a decrease in maximum efficiency of PSII (Fv/Fm) was also among the first detectable physiological responses to *Phytophthora* infections in this trial. Low values of Fv/Fm are considered signs of stress and were detectable two weeks after inoculation with these pathogens, being especially obvious in *P. plurivora*-infected plants which displayed a sharp dropping of values. Similar responses were also reported in other studies of *Phytophthora*-infested plants [35,39,60]. Furthermore, alterations in Fv/Fm caused changes in photosynthetic pigment composition as demonstrated by the values of the applied spectral indices (related to chlorophyll and carotenoids), which showed the most severe decrease in *P. plurivora*-infected plants compared to the control group. A decrease in Fv/Fm can also imply disturbances in the photosystem II electron transport chain, with negative consequences for photosynthetic performance (i.e., photoinhibition) [39]. However, due to the lack of data related to net photosynthesis the occurrence of photoinhibition could not be proven in our trial. Noteworthy, other authors observed similar reductions in maximal efficiency of PSII in one-year-old *Quercus ilex* seedlings infected by *P. cinnamomi* but only 30- and 90-days post-inoculation [61]. Similarly, a trial on *Persea americana* rootstocks inoculated with *P. cinnamomi* also showed later differences in Fv/Fm relative to control in some infected rootstock varieties [36]. In contrast to these studies, maximal photochemical efficiency of PSII could be used as an early indicator of stress caused by the root infections in the present study. Moreover, the lower Fv/Fm in *P. plurivora* and in *P. ×cambivora* A2-infected plants were accompanied by a decrease in the stomatal conductance after two weeks or in case of *P. ×cambivora* A2 after three weeks. In addition, reduced stomatal conductance was observed for *P. ×cambivora* A1 two weeks after inoculation. This stomatal closure can be related to the increase in sugar concentration in the leaves of plants infected with these pathogens, as observed in *P. cinnamomi*-inoculated chestnut plants [62] and can also be associated with the root necroses. A high concentration of sugars in leaves, particularly glucose and sucrose, has negative impacts on photochemical quenching by the inhibition of the Calvin cycle [63], which indirectly influences stomatal regulations. Results from underbark inoculation trials of *Alnus glutinosa* with *P. ×alni*, indicated that stomatal closure was not a consequence of reduced water potentials and hydraulic conductance but resulted from photosynthesis inhibition due to leaf starch accumulation since the translocation of assimilates to the root through the pathogen-infected necrotic phloem tissue was severely reduced [64]. In the present study, the decrease of both the concentration and relative abundance of sucrose in the roots of infected plants supports this hypothesis, as sucrose is the main form of transported carbohydrate [65]. If the decrease in carbon assimilation is strong enough, it can lead to the absorption of more radiation than can be thermally dissipated, and the production of reactive oxygen species, damaging the PSII as a consequence [66]. Moreover, the observed root rot in the *Phytophthora*-infected plants might have decreased water uptake and transport contributing to the observed stomatal limitations, especially in the third week when the root destruction was already very advanced, and mortality was high. However, several authors point out the involvement of other mechanisms such as a hypersensitive response (HR) [39], hormone signals [67,68] and either a chemical signaling or a higher internal CO_2_ concentration in the stomatal regulation [69] associated with root necrosis. It is likely that there is not a single general model which explains all physiological adjustments and we might expect different regulation mechanisms depending on the specific *Phytophthora*-host pathosystem. Furthermore, different experimental conditions could cause a different range of possible responses [70]. 

Metabolomics revealed alterations in both primary and secondary metabolites of *F. sylvatica* seedlings caused by *Phytophthora* infections. The deterioration of fine roots due to pathogen attacks is often accompanied by damage of cell membranes and the subsequent leakage of cell contents into the rhizosphere [71], in accordance with the significant decrease in most identified metabolites in the present trial. Furthermore, the pathogen certainly contributes to the decrease of metabolites, particularly sugars, by consuming them. Interestingly, leaves and roots showed an opposite response, with the leaves having an overall increase and the roots showing a decrease in the concentration of most metabolites. During the first hours of an infection, pathogens are usually able to hijack the host’s metabolism, modulating the transport of carbon and nitrogen resources towards the infected areas [72,73]. However, as the infection progresses the plant deploys countermeasures, which include not only the synthesis of protective metabolites [74], but also restrictions in the transport of photoassimilates towards the pathogen [75]. Therefore, the interpretation of metabolomics data in infected plants is complex, as it is difficult to determine whether the observed changes are a consequence of the attacking pathogen or the defensive actions of the host plant [73]. The decrease in the root metabolite pool observed in *F. sylvatica* seedlings was most likely a consequence of the combined action of both the pathogen, feeding on the plant’s primary metabolites [76], and the host, limiting the transport of photoassimilates from the shoot in order to starve the pathogen as demonstrated here by the increase of sugar contents in the leaves. Therefore, in agreement with [77], the most affected metabolic pathways in the present study were related to amino acid and sugar metabolism, and to the TCA cycle. 

Contrary to the overall decrease in metabolite concentration in roots of infected plants, two weeks after inoculation the concentration of several sugar alcohols, namely D-glucitol, glycerol and L-arabitol, was found to be either higher or, at least equal, to control plants and their relative abundance was higher. This may suggest that infected plants were favoring the synthesis or transport of these compounds, which can serve as osmoprotectans [78], over the other metabolites that were severely depressed as a reaction to the *Phytophthora*-caused root rot. However, certain sugar alcohols like L-arabitol have also been suggested to possess ROS-scavenging properties [79,80]. Therefore, it is possible that the relative increase in these compounds is not caused by the host, but by the pathogen attempting to fight the host’s defense mechanisms that rely on hypersensitive responses. This would be a role similar to that already established for mannitol, which is commonly associated with filamentous pathogen attack mechanisms [81].

Similarly, L-5-Oxoproline concentration increased in the roots of plants inoculated with *P. ×cambivora* A1 three weeks after inoculation. This could have been a reaction against the *Phytophthora*-root rot, since L-5-Oxoproline has been suggested to be a compatible solute with a role in osmoprotection [82]. Alternatively, this increase in L-5-Oxoproline, a product of glutathione degradation [83], could also indicate the increase in glutathione turnaround. Glutathione is a secondary metabolite with a demonstrated role as a response against *Phytophthora* infections [84]. In either case, the increase in L-5-Oxoproline seems to be indicative of a plant defense response. Interestingly, plants infected with *P. ×cambivora* A1 showed a higher survival rate than those infected with *P. plurivora* and, to a lesser extent, *P. ×cambivora* A2. 

In accordance with the results obtained from the physiological measurements, the metabolome of plants infected with *P. ×cambivora* A1 was also less affected compared to plants infected with *P. plurivora* and *P. ×cambivora* A2. One of the most interesting leaf metabolites was traumatic acid which showed higher concentrations in the leaves of plants infected with *P. ×cambivora* A2 and *P. plurivora* but did not increase after inoculation with *P. ×cambivora* A1. As it is commonly associated with biotic and abiotic stresses, in particular those involving physical damages [85,86], higher traumatic acid levels might indicate higher aggressiveness of *P. ×cambivora* A2 and *P. plurivora*. Similarly, concentrations of L-proline, a universal stress-related compound [87], also increased in the leaves of plants infected with *P. ×cambivora* A2 and *P. plurivora*. indicating increased stress resulting from the root infections [62]. 

## 5. Conclusions

The homothallic pathogen *P. plurivora* was more aggressive to young *F. sylvatica* plants than the A1 and A2 mating types of *P. ×cambivora* causing faster mortality. However, in terms of physiological and metabolomic responses, *P. plurivora*-infected plants did not differ from *P. ×cambivora* A2-infected plants. Apparently, aggressive pathogens with fast colonization of host tissues such as *P. plurivora* would require physiological and metabolomic measurements at shorter time intervals to detect differences. The A1 and A2 mating types of *P. ×cambivora* caused similar mortality rates. However, *P. ×cambivora* A2 had significantly stronger effects on the metabolome than *P. ×cambivora* A1 which might indicate behavioral differences between both mating types resulting in subtle differences in the beech seedlings adjustments. Moreover, *P. ×cambivora* A1 showed delayed sporulation on the infected roots with sporangia produced mainly in the third week of the trial. More research is needed to determine differences in the aggressiveness of the A1 and A2 mating types of *P. ×cambivora*, including a larger range of strains and, therefore, of genotypic diversity. 

## Data Availability

The data presented in this study are available on request from the corresponding author.

## Supplementary Materials

The following supporting information can be downloaded at: https://www.mdpi.com/article/10.3390/jof8030298/s1, Figure S1: Mean physiological values of *Fagus sylvatica* seedlings infected with the isolate TJ 71 of *P. plurivora*, the isolates TJ30 and TJ543 of A1 mating type of *P. ×cambivora* and the isolates TJ29 and TJ197 of A2 mating type of *P. ×cambivora* and of non-infected control seedlings: Mean values of spectral reflectance calculated with the Normalized Difference Vegetation Index (NDVI), the Photochemical Reflectance Index (PRI), Structure Intensive Pigment Index (SIPI) and the Carotenoid Reflectance Index (CRI1), mean values of chlorophyll fluorescence expressed as maximum quantum yield of photosystem II and stomatal conductance values (gs) of the seedlings two and three weeks after inoculation.; Table S1: Relative abundance (%) of total amino acids, total organic acids, total sugars, and selected metabolites in the leaves and roots of *Fagus sylvatica* plants infected with *P. plurivora* and the A1 and A2 mating types of *P. ×cambivora* and of non-infected control seedlings 2 and 3 weeks after inoculation.
